# Angiogenesis Interactome and Time Course Microarray Data Reveal the Distinct Activation Patterns in Endothelial Cells

**DOI:** 10.1371/journal.pone.0110871

**Published:** 2014-10-16

**Authors:** Liang-Hui Chu, Esak Lee, Joel S. Bader, Aleksander S. Popel

**Affiliations:** 1 Department of Biomedical Engineering, School of Medicine, Johns Hopkins University, Baltimore, Maryland, United States of America; 2 High-Throughput Biology Center, Johns Hopkins University, Baltimore, Maryland, United States of America; 3 Department of Oncology and Sidney Kimmel Comprehensive Cancer Center, School of Medicine, Johns Hopkins University, Baltimore, Maryland, United States of America; Center for Cancer Research, National Cancer Institute, United States of America

## Abstract

Angiogenesis involves stimulation of endothelial cells (EC) by various cytokines and growth factors, but the signaling mechanisms are not completely understood. Combining dynamic gene expression time-course data for stimulated EC with protein-protein interactions associated with angiogenesis (the “angiome”) could reveal how different stimuli result in different patterns of network activation and could implicate signaling intermediates as points for control or intervention. We constructed the protein-protein interaction networks of positive and negative regulation of angiogenesis comprising 367 and 245 proteins, respectively. We used five published gene expression datasets derived from in vitro assays using different types of blood endothelial cells stimulated by VEGFA (vascular endothelial growth factor A). We used the Short Time-series Expression Miner (STEM) to identify significant temporal gene expression profiles. The statistically significant patterns between 2D fibronectin and 3D type I collagen substrates for telomerase-immortalized EC (TIME) show that different substrates could influence the temporal gene activation patterns in the same cell line. We investigated the different activation patterns among 18 transmembrane tyrosine kinase receptors, and experimentally measured the protein level of the tyrosine-kinase receptors VEGFR1, VEGFR2 and VEGFR3 in human umbilical vein EC (HUVEC) and human microvascular EC (MEC). The results show that VEGFR1–VEGFR2 levels are more closely coupled than VEGFR1–VEGFR3 or VEGFR2–VEGFR3 in HUVEC and MEC. This computational methodology can be extended to investigate other molecules or biological processes such as cell cycle.

## Introduction

Angiogenesis, the formation of new blood vessels from pre-existing vessels, is involved in both physiological (e.g. development, wound healing and exercise) and pathological conditions (e.g. cancer and ocular neovascularization, such as neovascular age-related macular degeneration). Numerous molecules are involved in angiogenesis: for example, vascular endothelial growth factors (VEGF) and their receptors, fibroblast growth factors (FGF) and their receptors, proteins in the matrix metalloproteinase (MMP) and Notch families. Other pro-angiogenic factors such as angiopoietin-1 and anti-angiogenic factors such as thrombospondin-1 are also associated with regulation of angiogenesis. In order to integrate hundreds of angiogenesis-related molecules and infer angiogenesis-annotated genes, we have developed an algorithm to construct the angiome, a global protein-protein interaction network (PIN) relevant to angiogenesis [1].

Major regulators of angiogenesis for the endothelial cell, both ligands and their cell-surface receptors, were summarized in [2]. These regulators were classified as pro- or anti-angiogenic; such classification is important for application of our understanding of angiogenesis regulation to diseases. For example, suppression of major angiogenic regulators like VEGFA (conventionally referred to as VEGF), or release of endogenous anti-angiogenic factors like endostatin or thrombospondin can be used to inhibit tumor angiogenesis. An extended list of molecules involved in regulation of angiogenesis was constructed in [1], which included the families of VEGF, TGF (transforming growth factor), IGF (insulin-like growth factor), and PDGF (platelet-derived growth factor). Negative regulators of angiogenesis and associated proteins, including chemokines, angiopoietin, and serpin, were also considered.

Time course microarray data can help identify genes that are important in angiogenesis [1], [3]. Cultured endothelial cells are widely used in angiogenesis research. The most commonly used EC are human umbilical vein EC (HUVEC) and human microvascular EC (MEC); telomerase-immortalized human microvascular (TIME) EC are also used in functional genomics angiogenesis research [4]. Several time course microarray studies have been conducted to identify expressed genes in VEGF-treated HUVEC [5], MEC [6] and TIME cells [7]. The goal of this study is to combine the angiome with time-series gene expression data on VEGF-treated EC to investigate the dynamic responses of the key proteins and protein complexes in angiogenesis under different in vitro experimental conditions.

## Materials and Methods

### Constructing the networks of positive and negative regulation of angiogenesis

**Figure 1:**
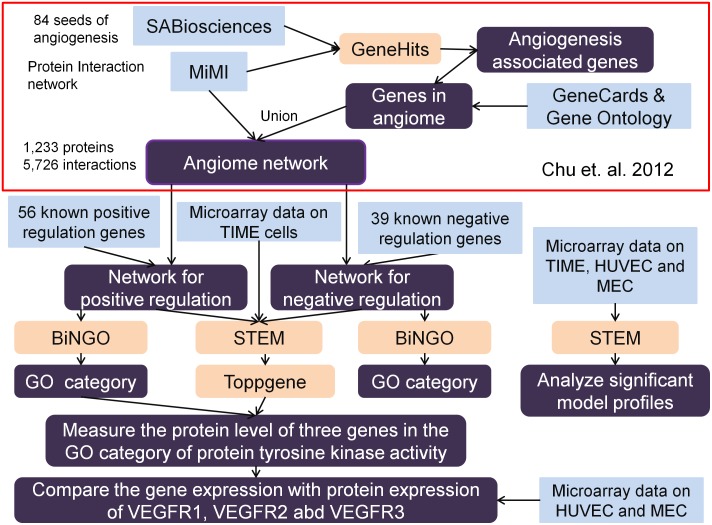
Flowchart of finding the protein complexes of angiome and merging time course gene expression data. We marked the methods used in the angiome study [1] with the red frame, and displayed the new methods in the lower part of the figure. These new strategies used in this study include software such as BiNGO (Biological Networks Gene Ontology) and STEM (Short Time-series Expression Miner), curated gene sets of positive and negative regulation of angiogenesis, use of microarray datasets and experimental design.

The flowchart of constructing the PIN of positive and negative regulation of angiogenesis is shown in Figure 1. We have constructed a gene search engine GeneHits described in [1] (accessible at http://sysbio.bme.jhu.edu). We constructed the angiome (the global protein-protein interaction network of angiogenesis) using the resources of SABiosciences, Gene Ontology (GO) and GeneCards [8]. The information on edges was downloaded from Michigan Molecular Interactions (MiMI) [9], which integrates eleven protein interaction data sources (BIND, CCSB, DIP, GRID, HPRD, IntAct, KEGG, MDC, MINT, PubMed and Reactome). The angiome network comprises 1,233 proteins and 5,726 interactions [1]. We will describe the new strategies, software and experimental datasets used in this study in the following sections.

Gene Ontology (GO) provides a rich resource of gene functions and locations in many different species [10]; positive regulation of angiogenesis (GO:0045766) and negative regulation of angiogenesis (GO:0016525) are included. Four genes are listed in both positive and negative regulators of angiogenesis: thrombospondin 1 (THBS1), angiopoietin 4 (ANGPT4), chemokine receptor 1 (CX3CR1), and serpin peptidase inhibitor member 1 (SERPINE1). However, THBS1 and SERPINE1 have been identified as anti-angiogenic [11]–[13]. Angiopoietin ANGPT4 is a protein that promotes angiogenesis [14]. Fractalkine (FKN)-induced activation of CX3CR1 in EC leads to in vivo angiogenesis through the induction of HIF-1alpha and VEGF-A gene expression by CX3CR1 activation and subsequent VEGF-A/KDR-induced angiogenesis [15]. Table 1 (A) and (B) presents 56 and 39 proteins annotated as positive and negative regulation of angiogenesis, respectively. We select the proteins in the extended angiome [1] which are linked to the 56 and 39 proteins in Table 1 (A) and (B) and their interactions to construct the two networks of positive and negative regulation of angiogenesis, respectively. Cytoscape is used to draw the PIN [16].

**Table 1:** List of genes in the angiome that are annotated as positive and negative regulators of angiogenesis shown in (A) and (B), respectively.

(A) 56 proteins annotated as positive regulators of angiogenesis: ADM, AGGF1, ANGPT4, ANGPTL3, ANXA3, AQP1, BTG1, C3, C3AR1, C5, CCL11, CCL24, CCL5, CCR3, CD34, CHRNA7, CTSH, CX3CR1, EPHA1, ERAP1, F3, FGF1, FGF2, FLT1, GATA2, GATA4, GATA6, HDAC9, HIF1A, HIPK1, HIPK2, HMOX1, IL1A, IL1B, KDR, MMP9, NOS3, PRKD1, PRKD2, PTGIS, PTGS2, RAMP2, RAPGEF3, RHOB, RRAS, RUNX1, SFRP2, SPHK1, TEK, TNFRSF1A, TNFSF12, TWIST1, UTS2R, VEGFA, VEGFB, WNT5A
(B) 39 proteins annotated as negative regulators of angiogenesis: AMOT, ANGPT2, APOH, BAI1, CCL2, CCR2, COL4A2, COL4A3, CXCL10, FASLG, FOXO4, GHRL, GTF2I, HDAC5, HHEX, HOXA5, HRG, KLF4, KLK3, KRIT1, LECT1, LIF, MAP2K5, NF1, NPPB, NPR1, PDE3B, PF4, PML, PTPRM, ROCK1, ROCK2, SERPINE1, SERPINF1, STAB1, THBS1, THBS2, THBS4, TIE1

### Microarray data analysis

We compiled five time-course microarray datasets at different experimental conditions on endothelial cells (Table 2). Schweighofer *et al.* [5] measured gene expression in HUVEC stimulated by VEGF and epidermal growth factor (EGF) (GSE10778). Glesne *et al.* [6] measured transcripts during proliferation and tubulogenesis in human MEC stimulated with VEGF (GSE2891). Mellberg *et al.* [7] cultured TIME cells (telomerase-immortalized human microvascular endothelial cells) in 3D collagen gels and on 2D fibronectin matrix, stimulated with VEGF and measured gene expression. Raw microarray data on TIME cells from Mellberg *et al.* [7] were kindly provided by the authors. We downloaded the time course microarray datasets from Gene Expression Omnibus (GEO) databases [5], [6] and recovered the missing data from Mellberg *et al.* [7] using GenePattern 3.6.1 [17]. Gene Expression Omnibus (GEO) data were imported by GEOImporter version 5. Genes with missing values in Mellberg *et al.* [7] were recovered by the *k* nearest neighbors (KNN) algorithm in ImputeMissingValuesKNN version 13 module. We used the default settings in GenePattern software.

**Table 2:** Five VEGF-treated time-course microarray datasets with different experimental conditions on endothelial cells.

Treatment	Cells	Time	Resource	Ref
VEGFA	HUVEC	0,0.5,1,2.5,6 hr	GSE10778	(Schweighofer, et al., 2009)
VEGFA	MEC(proliferation)	0,0.5,1,2,4 hr	GSE3891	(Glesne, et al., 2006)
VEGFA	MEC(tubulogenesis)	0.5,1,2,4,8 hr	GSE3891	(Glesne, et al., 2006)
VEGFA	TIME (3Dcollagen I)	15 min,1,3 6,9,12, 18,24 hr	Provided by authors	(Mellberg, et al., 2009)
VEGFA	TIME (2Dfibronectin)	15 min,1,3,6,9,12, 18,24 hr	Provided by authors	(Mellberg, et al., 2009)

### Temporal expression pattern

We use Short Time-series Expression Miner (STEM) [18] to identify significant temporal expression profiles and the genes associated with these profiles integrated with Gene Ontology (GO) database from microarray experiments. The clustering method of gene expression profiles is based on STEM clustering method; details of the algorithms are described in [19]. This clustering algorithm first selects several distinct and representative temporal expression profiles, called “model profiles”. The model profile starts at the first time point, and then the profile between the two time points can be unchanged, increase or decrease with an integer number of time units. The model profiles are selected independently from the data to determine the significance of the different clusters. The STEM clustering algorithm assigns each gene to the model profile that matches the expression profile of genes most closely by the correlation coefficient. We set GO annotations as biological processes and molecular functions with minimum GO depth of 3, number of permutations per gene to 50, and significance level p-values to 0.05 by Bonferroni correction.

### Functional enrichment of genes associated with positive and negative regulation of angiogenesis

We used BiNGO 2.44 (Biological Networks Gene Ontology tool) [20] on Cytoscape 2.8 [16] for the functional enrichment analysis of genes in the positive and negative regulation of angiogenesis PINs to identify pathways and biological processes. The p-values were computed by the hypergeometric test, and the Benjamini & Hochberg false discovery rate (FDR) correction was also computed at a significance level 0.05.

### Cell culture

Human microvascular endothelial cells (MEC) and human umbilical vein endothelial cells (HUVEC) were purchased from Lonza (Walkersville, MD). MEC were propagated in microvascular endothelial cell growth medium-2 (EGM-2MV, Lonza). HUVEC were grown in endothelial cell growth medium-2 (EGM-2, Lonza). Cells were maintained under standard conditions of 37°C and 5% CO_2_ and the passage numbers of the endothelial cells were kept between 3 and 6.

### Western blot assay

MEC and HUVEC in passages 3 to 6 (Lonza) were plated in 75T tissue culture flasks at 1,000,000 cells/well in the normal growth media (EGM-2MV for MEC; EGM-2 for HUVEC, from Lonza). After 48 hr, normal growth media were replaced with serum-free media (EBM-2 without supplements) and incubation lasted 24 hr to starve the cells. Human VEGF_165_ (50 ng/ml, R&D systems) in serum-free media was applied, and the flasks were incubated for 0, 1, 3, 6, 12, 24 hr at 37°C, and 5% CO_2_. VEGF treatment was stopped by adding cold PBS and the cells were lysed in cold lysis buffer (150 mM NaCl, 1 mM EDTA, 1 l/ml protease inhibitors (Sigma Aldrich), 1 l/ml phosphatase inhibitors (Sigma) and 1% Triton X-100) for 2 hr at 4°C, then scraped to collect the lysates. Cell lysates were spun at 14,000 g for 30 min to remove dead cells and cell debris. Cell lysates were separated by sodium dodecyl sulfate polyacrylamide gel electrophoresis (SDS-PAGE) and transferred to nitrocellulose blots (Invitrogen, Carlsbad, CA), using the iBlot transfer module (Program 3, 14 min). We blocked the nitrocellulose membrane for 1 hr with 5% non-fat milk+1% BSA (bovine serum albumin, Sigma) in TBST (1X TBS with 0.1% Tween 20) at room temperature, and the membrane was probed with antibodies detecting human VEGFR1 or VEGFR2 or VEGFR3 at 1∶1000 dilution (Cell Signaling Technology and Abcam). Glyceraldehyde 3-phosphate dehydrogenase (GAPDH, 1∶2000, Cell Signaling) was used as a loading control. HRP-labelled secondary antibodies were added at 12000 dilution and protein bands were detected with the Amersham ECL Prime Western Blotting Detection Reagent (GE Healthcare). Western bands were analyzed by quantifying number of pixels in the band using ImageJ free software (NIH, Bethesda, MD). Full length VEGFR1 (180 kDa) in MEC and HUVEC were analyzed, as HUVEC does not show sVEGFR1 (110 kDa). Similarly full length VEGFR3 (195 kDa) was analyzed, as HUVEC do not show the unglycosylated precursor form (175 kDa). Each band was normalized by using the GAPDH level.

## Results

### Constructing the networks of positive and negative regulation of angiogenesis

Following the description in Methods and flowchart in Figure 1, we constructed the two networks of positive and negative regulation of angiogenesis. The PIN of positive regulation of angiogenesis comprises 367 proteins and 1,972 interactions (Table S1); the PIN of negative regulation of angiogenesis comprises 245 proteins and 1,154 interactions (Table S2). Some proteins in the positive regulation of angiogenesis are also connected to the proteins in the negative regulation of angiogenesis by physical interactions present in the MiMI [20] database and literature reports, such as anti-angiogenic thrombospondin (THBS1) directing binding to angiogenic proteins COL1A1 (collagen type I) [21] and MMP9 (matrix metallopeptidase 9) [22]. Details of protein interaction types and resources of interactions are provided in Table S1 and S2. The list of repeated proteins included in both positive and negative regulation of angiogenesis is provided in Table S2. We used BiNGO 2.44 (Biological Networks Gene Ontology tool) [23] for the functional enrichment of genes in the two angiogenesis PINs (Table S3 and S4.).

### Temporal gene expression pattern

Among microarray datasets shown in Table 2, Mellberg’s dataset on TIME cells [7] contains the most time points at 15 min and 1, 3, 6, 9, 12, 18, and 24 h. We used the STEM [18] to identify significant temporal gene expression profiles and the genes associated with these profiles integrated GO database. We found the temporal gene expression pattern of all the genes in the raw microarray data. We normalized the microarray data to the first time point in each of the set [5], [6] except Mellberg’s data [7] which have been normalized to the untreated conditions. The maximum number of model profiles was set as 20 and also compared with the maximum number of model profiles as 10 and 40 in temporal gene expression profiles of TIME cells on 2D fibronectin and 3D collagen I (Table S5). The genes with absolute log2 fold change between the maximum and minimum values of any two over all time points less than 1 are removed in the analysis.

**Figure 2:**
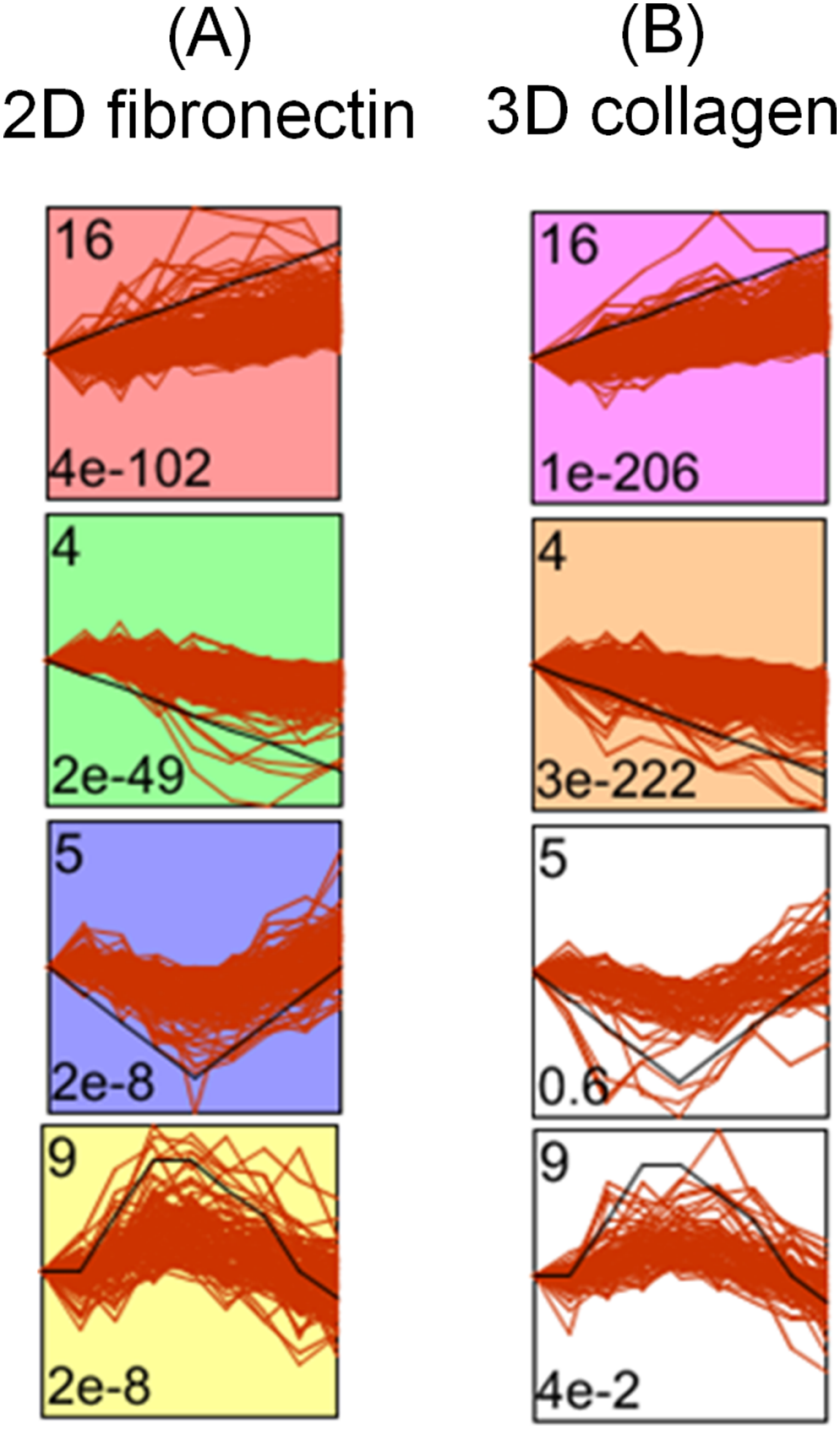
Temporal gene expression profiles on 2D fibronectin and 3D collagen I for TIME cells in (A) and (B), respectively.

We show the four statistically significant (adjusted p-value<0.05 by Bonferroni correction) temporal gene expression profiles of TIME cells on 2D fibronectin, and sort the four profiles by their p-values in Figure 2 (A). The p-value was calculated by the number of genes assigned to the model profile, compared to the expected number of assigned genes. The number on top left represents the assigned profile number by STEM, and the number on bottom left represents the significance level before the Bonferroni correction. The box is colored if the statistically significant number of genes, based on the adjusted p-value<0.05 by Bonferroni correction, are assigned to the model profile. The black and red lines in the individual profile boxes indicate the assigned pattern, e.g. the sequence (0,1,2,3,4,5,6,7,8) over the eight time points and initial points in profile #16, and the gene expression of genes assigned in that profile. We compare the four statistical significant profiles on 2D fibronectin in Figure 2 (A) with 3D type I collagen, and plot the four profiles (#16, #4, #5, #9) in Figure 2 (B). We found statistically significant patterns of continuous up- and down-regulation depicted by profiles #16 and #4 (shown on the top-left corner of each profile box) exist for both matrices on TIME cells, but fluctuation patterns depicted by profiles #5 and #9 are only exhibited on 2D fibronectin (Figure 2A).

We found that the top five most significant GOs on 2D fibronectin of profile #16 (Bonferroni corrected p-value<0.008 by using a randomization test) in Figure 2A are angiogenesis (GO:0001525), vasculature development (GO:0001944), extracellular matrix organization (GO:0030198), extracellular structure organization (GO:0043062) and system development (GO:0048731). The top five most significant GOs (corrected p-value<0.001) on 3D type I collagen matrix of the monotonically increasing profile #16 in Figure 2B are angiogenesis (GO:0001525), blood vessel morphogenesis (GO:0048514), vasculature development (GO:0001944), blood vessel development (GO:0001568) and extracellular matrix organization (GO:0030198). We found several significant GOs with similar GO functions, such as angiogenesis (GO:0001525), vasculature development (GO:0001944), blood vessel development (GO:0001568), and blood vessel morphogenesis (GO:0048514). The clusters of significant profiles, GOs and genes in the profile are listed in Table S6.

**Figure 3:**
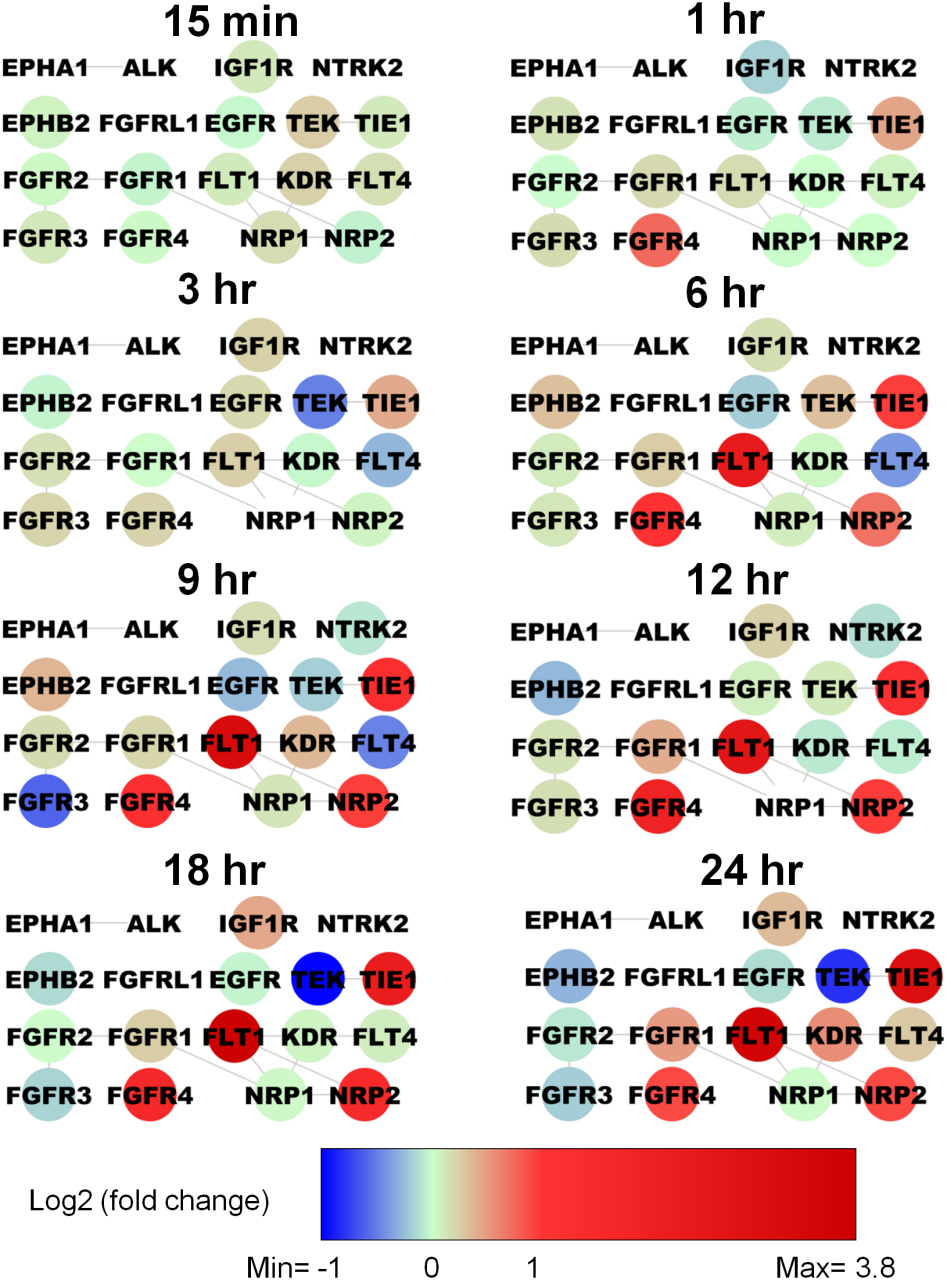
Activation pattern of receptor tyrosine kinases on 3D collagen I for TIME cells.

We compared the statistically significant model profiles between 2D fibronectin (#16, 4, 5 and 9 in Figure 2A) and 3D type I collagen (#4 and 16 in Figure 2B) by the maximum uncorrected intersection p-value as 0.005 in Table S7. Table S7 also lists the genes in the pairwise comparisons with at least one significant GO category by the corrected p-value<0.05. The p-value was calculated based on the hypergeometric distribution of the intersection of genes assigned to the two profiles, one profile from the original data set and the other from the comparison data set [18]. 115 and 112 genes are assigned to the intersection of the increasing profile #16 and the decreasing profile #4 on two matrices, respectively. Among these 115 genes in pattern #16 of the intersection, fourteen genes annotated as GO:0001525 angiogenesis (corrected p-value = 0.004) include ADM, CDH13, COL4A1, EPHA2, HSPG2, ISL1, ITGAV, MMP2, NOTCH4, RAMP2, RGC32, RHOB, VASH1 and VEGFC. We found three genes, the LIM-homeobox transcription factor islet-1 (ISL1), Response gene to complement 32 (RGC32), and vasohibin-1 (VASH1) annotated as angiogenesis, which were not included in the angiome in our previous study [1]. We also compare the different activation pattern pairs between the two substrates. Profile #5 on 2D fibronectin and #4 in 3D collagen I share some genes in the GO:0048584 positive regulation of response to stimulus (corrected p-value = 0.006), including BCAR1, DAB2, DAPK3, DBNL, DUSP7, F2RL1, FZD4, GPR177, IGKC and TNFSF10.

### Significant temporal gene expression profiles in VEGFA-treated MEC and HUVEC

We used the STEM software [18] to find significant temporal gene expression profiles in previously reported datasets of VEGFA-treated MEC (GSE3891) [6] and HUVEC (GSE10778) [5]; the results are presented in Tables S8–S9 listing the significant profiles (p-value<5E-2). The GOs for the significantly decreasing profiles #4 (p-value = 6.40E-40) in VEGF-treated MEC during proliferation [7] include translational initiation, termination and elongation. We compare the genes in the intersection of significant profiles during proliferation and tubulogenesis in VEGFA-treated MEC (Table S8). The GO categories for the genes in the intersection of increasing profile #17 during tubulogenesis and decreasing profile #4 during proliferation include translational termination and elongation (p-value<0.001). This analysis shows that some genes involved in protein translation behave differently during endothelial cell proliferation and tubulogenesis. The temporal gene expression profiles in HUVEC [5] in Table S9 show more diverse patterns than TIME cells shown in Figure 2.

### Activation patterns of the receptor protein tyrosine kinase

We used the defined gene set of the 367 and 245 genes in the positive and negative regulation of angiogenesis in STEM clustering, respectively (Table S10). We found 21 and 19 genes in the positive regulation of angiogenesis which were assigned to the increasing profile #16 on 3D type I collagen and 2D fibronectin, respectively. We further used Toppgene [24] to analyze the functional enrichment of proteins included in the increasing profile #16 of positive regulation of angiogenesis (Table S10). One of the top significant GO molecular functions for 21 and 19 genes in the increasing profile #16 of positive regulation of angiogenesis on 3D type I collagen is protein tyrosine kinase activity (p-value = 9.568E-5 and 2.648E-3).

Since tyrosine kinase activity is of great interest in translational applications, we scrutinize the genes annotated as protein tyrosine kinase activity in the functional enrichment of positive regulation of angiogenesis in Table S3. The GO category “transmembrane receptor protein tyrosine kinase activity” (adjusted p-value = 1.64E-13) contains eighteen proteins ALK, EGFR, EPHA1, EPHB2, FGFR1, FGFR2, FGFR3, FGFR4, FGFRL1, FLT1 (VEGFR1), FLT4 (VEGFR3), IGF1R, KDR (VEGFR2), NRP1, NRP2, NTRK2, TEK and TIE1. We merge proteomic and genomic data based on the 2007 protocol [25] by Cytoscape [16]. The proteins with gene transcripts on 3D type I collagen for TIME cells in Figure 3 show that FLT1 is activated consistently after 6 h, KDR only activated at 24 h, and FLT4 decreased from 15 min to 9 hr then increased from 12 hr to 24 hr. The VEGF ligands family and their receptors play important roles in the development, maintenance, and remodeling of the vasculature [26], [27]. Thus, we select three VEGF receptor tyrosine kinases VEGFR1 (FLT1), VEGFR2 (KDR), and VEGFR3 (FLT4) to perform protein-level time series in vitro experiments.

### Comparison between gene transcripts and protein expression

**Figure 4:**
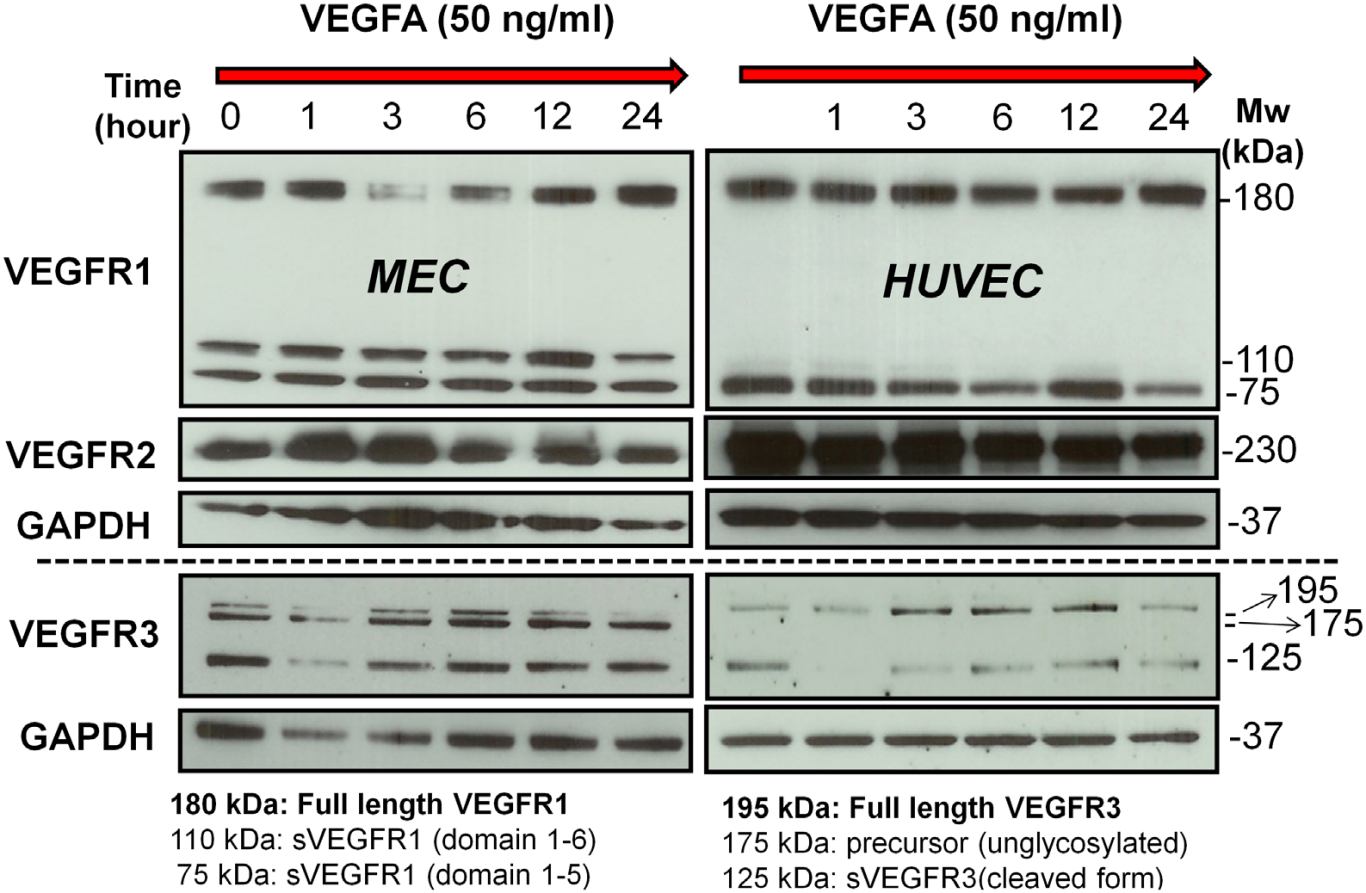
Experiments of VEGFR1, VEGFR2 and VEGFR3 for MEC and HUVEC.

We explored time-dependent VEGF receptor expression in blood endothelial cells after VEGF treatment, as the VEGF-VEGFR axis is pivotal in endothelial cell growth and maintenance. The experimental results for FLT1 (VEGFR1), KDR (VEGFR2) and FLT4 (VEGFR3) are shown in Figure 4. Briefly, one million of HUVEC or MEC were starved overnight, after which we treated the cells with 50 ng/ml of human VEGF_165_ and incubated them for 0, 1, 3, 6, 12 and 24 hr at 37°C. Total protein levels of VEGFR1/2/3 and glyceraldehyde 3-phosphate dehydrogenase (GAPDH) were obtained for normalization in data analyses. The number of pixels of each western band was analyzed using ImageJ (NIH, Bethesda). In VEGFR1 and VEGFR3 analyses, the full length VEGFR1 (180 kDa) and the full length VEGFR3 (195 kDa) in MEC and HUVEC were analyzed, as HUVEC do not express some isoforms. Each band was finally normalized by the GAPDH level.

**Figure 5:**
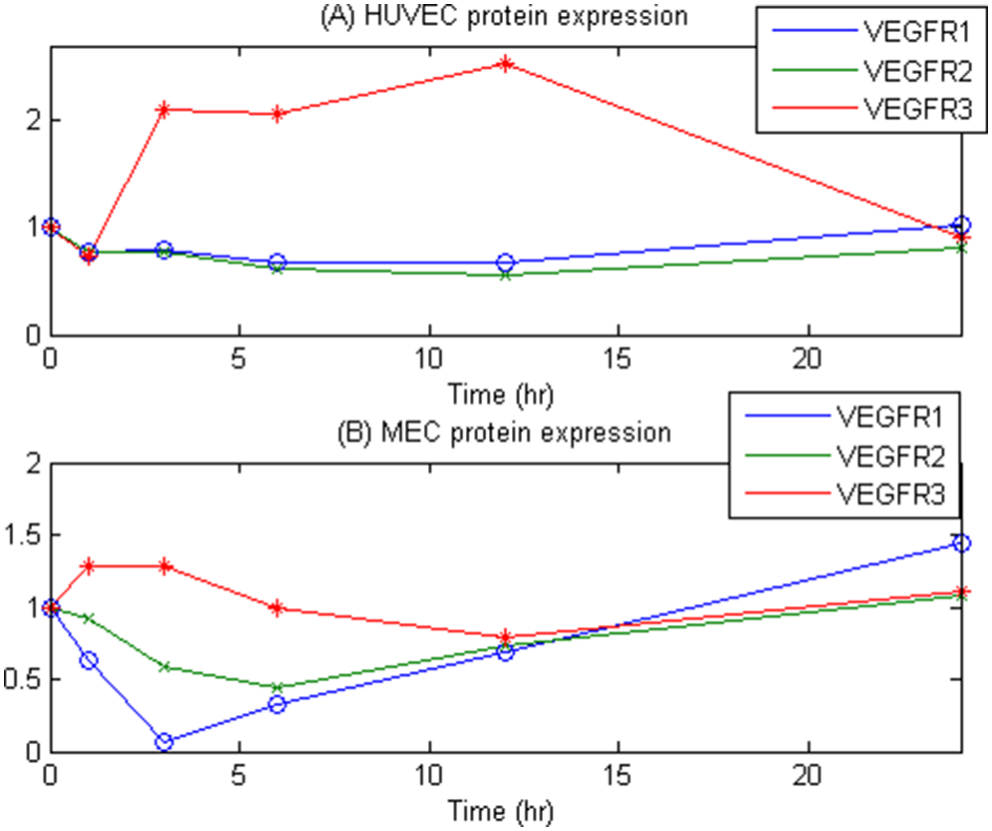
Normalized protein level measurement of VEGFR1, VEGFR2 and VEGFR3 to GAPDH on HUVEC and MEC in (A) and (B), respectively.

We plotted the ratio of measured protein levels of VEGFR1, VEGFR2 and VEGFR3 to the GAPDH in HUVEC and MEC in Figure 5 (A) and (B), respectively, normalized to the first time point. We observed that VEGFR1 and VEGFR2 levels are more closely coupled than VEGFR1-VEGFR3 or VEGFR2-VEGFR3 in HUVEC and MEC. Interestingly, VEGFR1 and VEGFR2 were restored to the initial protein level after the mid-time point of VEGF treatment, suggesting that some downstream signaling of VEGF pathway may induce VEGFR1 and VEGFR2 expression. Figure 5 (B) shows that VEGFR1 drops after VEGF treatment in MEC, showing its minimum level at 3 hr, then, VEGFR1 recovers continuously. Similarly, VEGFR2 shows minimum level at 6 hr, and recovered after that time point. This “drop and recovery” pattern is not shown in VEGFR3. There might indicate different regulation mechanisms for VEGFR1/2 and VEGFR3 in endothelial cells.

VEGFR1 and VEGFR2 levels increase after 12 hr in VEGF treated MEC and HUVEC. This confirms that VEGF is a potent mitogen for blood endothelial cells, and that VEGF-treated HUVEC and MEC may not be significantly involved in VEGFR3 signaling, which is known as a lymphangiogenic receptor, though these endothelial cells express VEGFR3. Also crosstalk between VEGFR1 and VEGFR2 in the presence of VEGF, especially their reciprocal mitogenic signaling is an important topic in the endothelial cell biology. VEGFR1 has three isoforms: full length/membrane bound form (180 kDa), and two soluble forms (110, 75 kDa) [28]. These soluble VEGFR1 lack the intracellular tyrosine kinase and the transmembrane domains, and play as scavenger molecules of VEGF. VEGFR3 has three isoforms: glycosylated (195 kDa), unglycosylated precursor (175 kDa), and their cleaved form (125 kDa). Interestingly, HUVEC did not show larger soluble form of VEGFR1 (110 kDa) and unglycosylated precursor form of VEGFR3 (175 kDa) compared to MEC that express all three isoforms of VEGFR1 and VEGFR3 (Figure 4) [29]. Different expression of the VEGFR isoforms, their processes of cleavage, and biological functions of these isoforms under VEGF treatment in different endothelial cells need to be further investigated.

The study of time-specific differences in gene expression in EC could provide important insights into their role in normal physiology and diseases. In normal physiology, temporal gene expression can result in EC heterogeneity [30]. EC heterogeneity is observed in different organs and different stages of development [31]. Our study may enable understanding of angiogenesis processes in different location or stages of organs by identifying crucial genes in each context. We particularly identified time-specific activation patterns of genes in VEGF-treated TIME cells, HUVEC and MEC. VEGF is pivotal for life: if it is abolished, it results in the embryonic death and impaired tissue maintenance and regeneration [32]. As our study was based on microarray data, it could be applied to the experimental design with time-dependent quantitative RT-PCR to identify other genes that regulate EC proliferation and migration in the presence of VEGF. Our analyses, however, need to be further explored at protein expression levels. The time-dependent approach for the gene expression in angiogenesis is also important for diseases. Plasma concentrations of VEGF and its receptors vary in a time-dependent manner before and after lung cancer surgery [33]. Proangiogenic plasma alterations such as Angiopoietin-1 (Ang1), VEGF and soluble VEGFR1 may result in cancer patients developing recurrent disease after surgery. Time-dependent changes of plasma VEGF levels and VEGFR1 in acute lung injury in the rat sepsis model revealed the pulmonary VEGF and the signaling pathways [33]. The VEGF and VEGFR1 levels are increased in liver tissues in lipopolysaccharide (LPS)-induced endotoxemia in a time-dependent manner [34]. Therefore, studies in VEGF-dependent diseases and associated abnormalities in blood endothelium can benefit from the current study.

In summary, we investigated the different activation patterns of genes in VEGF treated human endothelial cells (TIME cells, HUVEC and MEC). This computational methodology can be extended to investigate various VEGF dependent biological processes. All the files including the Cytoscape and microarray datasets for STEM simulations are provided on our laboratory website http://pages.jh.edu/~apopel/software.html.

## Conclusions

Combining gene expression data and protein interactions could reveal the dynamics of positive and negative regulation of angiogenesis in different endothelial cells and under different experimental conditions. We constructed two protein interaction networks representing positive and negative regulation of angiogenesis and found several clusters from gene ontology annotations and network properties. These findings capture the dynamics of protein interactions in regulation of angiogenesis, and can serve as a guide for experimental design related to activation patterns of important proteins in angiogenesis.

## Supporting Information

Table S1 **Proteins and interactions in positive regulation of angiogenesis.**
(XLS)

Table S2 **Proteins and interactions in negative regulation of angiogenesis.**
(XLS)

Table S3 **Functional enrichment analysis of genes in the positive regulation of angiogenesis.**
(XLS)

Table S4 **Functional enrichment analysis of genes in the negative regulation of angiogenesis.**
(XLS)

Table S5 **Comparison of number of model profiles as 10, 20 and 40 in STEM analysis.**
(XLSX)

Table S6 **Significant temporal profiles on 2D fibronectin and 3D type I collagen for TIME cells.**
(XLS)

Table S7 **Comparisons of significant profiles in 2D fibronectin and 3D type I collagen.**
(XLS)

Table S8 **Significant temporal profiles for MEC.**
(XLSX)

Table S9 **Significant temporal profiles for HUVEC.**
(XLSX)

Table S10 **Significant temporal profiles by defined gene sets of positive and negative regulation of angiogenesis in 2D fibronectin and 3D type I collagen.**
(XLSX)
